# Target engagement imaging of PARP inhibitors in small-cell lung cancer

**DOI:** 10.1038/s41467-017-02096-w

**Published:** 2018-01-12

**Authors:** Brandon Carney, Susanne Kossatz, Benjamin H. Lok, Valentina Schneeberger, Kishore K. Gangangari, Naga Vara Kishore Pillarsetty, Wolfgang A. Weber, Charles M. Rudin, John T. Poirier, Thomas Reiner

**Affiliations:** 1 Department of Radiology, Memorial Sloan Kettering Cancer Center, New York, NY 10065 USA; 2 Department of Chemistry, Hunter College and PhD Program in Chemistry, The Graduate Center of the City University of New York, New York, NY 10018 USA; 3 Department of Radiation Oncology, Memorial Sloan Kettering Cancer Center, New York, NY 10065 USA; 4 Molecular Pharmacology Program, Memorial Sloan Kettering Cancer Center, New York, NY 10065 USA; 5 Department of Radiology, Weill Cornell Medical College, New York, NY 10065 USA; 6 Department of Medicine, Memorial Sloan Kettering Cancer Center, New York, NY 10065 USA

## Abstract

Insufficient chemotherapy response and rapid disease progression remain concerns for small-cell lung cancer (SCLC). Oncologists rely on serial CT scanning to guide treatment decisions, but this cannot assess in vivo target engagement of therapeutic agents. Biomarker assessments in biopsy material do not assess contemporaneous target expression, intratumoral drug exposure, or drug-target engagement. Here, we report the use of PARP1/2-targeted imaging to measure target engagement of PARP inhibitors in vivo. Using a panel of clinical PARP inhibitors, we show that PARP imaging can quantify target engagement of chemically diverse small molecule inhibitors in vitro and in vivo. We measure PARP1/2 inhibition over time to calculate effective doses for individual drugs. Using patient-derived xenografts, we demonstrate that different therapeutics achieve similar integrated inhibition efficiencies under different dosing regimens. This imaging approach to non-invasive, quantitative assessment of dynamic intratumoral target inhibition may improve patient care through real-time monitoring of drug delivery.

## Abstract (web summary)

Treatment of small-cell lung cancer remains a challenge due to multiple mechanisms of resistance to current therapies; measuring patient response is crucial in adapting and choosing adequate treatment. Here the authors develop a strategy to visualise in vivo dynamics of a class of widely used PARP inhibitors.

## Introduction

While the diagnosis and treatment of many malignancies have seen significant improvements over recent decades^1^, the 5-year survival rates of small-cell lung cancer (SCLC), a subset of the general lung cancer population (13%, 29,000 of 221,000 patients in US annually), remain around 5% and are below 1% for the over 60% of patients that are diagnosed with extensive stage disease. The standard of care for advanced SCLC has essentially remained stagnant for more than 30 years.

The lack of progress, in part, can be attributed to the aggressive nature of this disease, which is exceptionally proliferative and rapidly develops resistance to chemotherapy. Therefore, novel treatment approaches are needed. Therapeutics that are targeted against a tumor specific biomarker have gained large interest because they can specifically act against tumor cells without the systemic toxicity and side effects of chemo- or radiotherapy. In the clinical reality, however, only a subset of patients responds well to targeted therapies. A better understanding of the spatial distribution and quantification of the target as well as the intratumoral drug-target interaction could strongly improve targeted therapy approaches by identifying particularly sensitive or resistant patient sub-populations, and by allowing ongoing monitoring of emerging resistance, enabling rapid change in chemotherapy regimens.

A radiolabelled, non-invasive imaging tracer would be an ideal candidate for such a diagnostic tool, because it would allow unlimited “sampling” of all metastases in an individual patient and provide contemporaneous uptake values, allowing quantitative measurements before, during and after treatment cycles.

One class of therapeutics that are being investigated as new treatment options for SCLC, are poly ADP-ribose polymerase (PARP) inhibitors. PARP inhibition, and the associated perturbation of the single-stranded DNA repair pathway, has been shown to be a promising therapeutic approach in both preclinical and clinical research settings^2^. The combination of PARP inhibitors and DNA damaging agents, such as temozolomide, has seen recent success and sufficient delivery of both drug classes potentiates their therapeutic effects^2,3^. One reason for this is that DNA damage repair plays an important role in the sensitivity of SCLC to chemotherapeutic agents, and consequently, current standard of care therapies for SCLC contain at least one DNA damaging agent. This sensitivity can be attributed in part to the genetics of this disease: nearly all patients have loss of the tumor suppressor genes *TP53* and *RB1*, which are critical to the normal function of multiple DNA damage response pathways and G1/S checkpoint maintenance, respectively^4,5^. Critically, there is not only consistent protein overexpression of PARP1 in SCLC, but also increased sensitivity to PARP1/2 inhibitors, in spite of intact BRCA^6^. On the basis of these observations and the underlying genetics of the disease, PARP inhibition is gaining considerable attention as a novel systemic treatment for SCLC (i.e., NCT02289690, NCT02734004, NCT01286987).

“One-size-fits-all” flat dosing regimens, or weight-based dosing regimens, have generally been used for members of this drug class, several of which have advanced to phase III trials. While this may be sufficient for some clinical applications, many patients – and in particular SCLC patients–may benefit from an immediate readout for determining the degree of on-target intratumoral PARP inhibition and, consequently, an imaging probe that can monitor and quantify PARP1/2 inhibition success.

In light of the expanding clinical relevance of PARP therapeutics, and the diversity of novel agents in this area, we became interested in exploring in vivo pharmacodynamic monitoring of PARP inhibitor target engagement in SCLC. A number of PARP therapeutics have entered late phase clinical research (talazoparib, veliparib) or are already FDA approved (olaparib, rucaparib, niraparib). While all of these small molecules possess unique pharmacokinetic profiles and therapeutic indices, they share one common binding motif: the NAD^+^ active site pocket of PARP. Therefore, while the development of an imaging tracer for each individual therapeutic would require considerable preclinical and clinical resources, we hypothesized that a single imaging tracer may be used to quantify target engagement for a broad group of PARP inhibitors, unlike other classes of drugs.

To test this hypothesis, we designed a series of experiments central to clinical translation of PARP target engagement imaging that address three fundamental questions: (1) What is the range of PARP expression in SCLC and will this range support quantitative assessment of PARP imaging? (2) Are the putative imaging reagents, [^18^F]PARPi and PARPi-FL, selective binders of PARP and do they have the same binding profile as their therapeutic counterparts? (3) Can target inhibition of PARP be quantified for PARP therapeutics generally and can our PARP imaging agents non-invasively predict target engagement in vivo?

We believe that finding and validating [^18^F]PARPi as a widely applicable, easy-to-use general PARP target engagement imaging agent is of high value for optimizing SCLC treatment. Such an agent wouldn’t just serve as a “companion imaging agent” for one individual molecularly targeted drug, but rather a whole class of therapeutics, dramatically expanding and amplifying its potential utility in clinical practice. Here using patient-derived xenograft models of SCLC, we present a highly sensitive and accurate technology for quantifying PARP inhibitors and target engagement in vivo in support of the clinical relevance of [^18^F]PARPi for treatment monitoring and prediction.

## Results

### PARP enzyme specificity of inhibitors and imaging agents

**Fig. 1:**
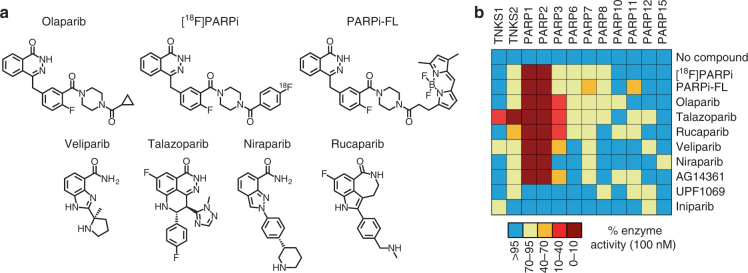
Properties of PARP inhibitors. **a** Structures of 5 clinical therapeutic PARP inhibitors and the 2 PARP imaging agents. **b** Heatmap visualizing enzymatic activity screening of 10 small molecules against 12 PARP enzymes at 100 nM concentration (for additional concentrations, see Supplementary Fig. 1)

PARP enzymes are a family of 17 proteins that share the same catalytic PARP domain^7^. PARP inhibitors have been shown to exhibit a wide range of pharmacologic specificity within this family^7^. For SCLC in particular, upregulation and overexpression of PARP1 has been demonstrated, and is proposed as a therapeutic target^6^. In developing PARP targeted imaging agents, we first wanted to ensure that the modifications made for imaging did not substantially alter target engagement with the key PARP enzymes. To determine the specificity of our imaging agents within this family of enzymes, and to compare their inhibition profile to clinically relevant PARP therapeutics (Fig. 1a), we conducted an enzyme activity inhibition screening across all available enzymes. Included in the study were five inhibitors in phase III clinical trials or FDA approved: olaparib, veliparib, talazoparib, niraparib, and rucaparib, together with our imaging agents [^18^F]PARPi and PARPi-FL. Besides [^18^F]PARPi and PARPi-FL, other PARP imaging agents have been reported in the literature^8^ and one of them was recently translated to the clinic^9^. We also included AG14361, (K_i_(PARP1) < 5 nM)^10^, UPF1069 (IC_50_(PARP2) = 0.3 ± 0.1 µM)^11^, and iniparib as negative control. We assessed the inhibitory activities of the PARP therapeutic agents and our imaging agents across 12 PARP enzymes including tankyrases TNKS1 and TNKS2 (Fig. 1b, Supplementary Fig. 1). The imaging agents generally exhibited comparable or higher specificity for PARP1/2 compared to the therapeutic molecules. At 100 nM, all therapeutic molecules, except niraparib, inhibited activity in PARP3 as well as PARP1/2 (>35% for PARP3, >90% for PARP1 and PARP2). Talazoparib also exhibited inhibition for both tankyrase enzymes, TNKS1 and TNKS2.^19^F-PARPi (the non-radioactive analog to [^18^F]PARPi) and PARPi-FL showed very low inhibition of TNKS1 and TNKS2 (≤5%) and PARP3 (≤30%). Inhibition of PARP1 and PARP2 was strong (both >90%), overlapping with the therapeutic clinical candidates and providing a rational basis for using our imaging agents as general sensors for PARP1/2 target engagement. Further comparison of all inhibitors used in this study (Table 1, Supplementary Fig. 2) showed similar binding affinities for all inhibitors including the imaging agents, with low nanomolar IC_50_ values reported for both PARP1 and PARP2. [^18^F]PARPi and PARPi-FL uptake therefore does not reflect expression of a single enzyme, but it is a sensor for how much of any tested PARP therapeutic has been retained in a given tissue, which makes using these imaging agents for target engagement imaging particularly appealing. This was also corroborated by the ability of PARPi-FL and [^18^F]PARPi to inhibit PARylation, similar to olaparib (Supplementary Fig. 3). One notable difference is in the trapping ability reported for each inhibitor, a mode of efficacy whereby the inhibitor traps the PARP enzyme at the site of DNA damage, leading to further DNA damage and ultimately cell death^12,13^. It has been reported that talazoparib is the most effective PARP trapping agent^14^, as well as the most potent PARP inhibitor in SCLC clinical testing^15^.

**Table 1:** Properties of PARP inhibitors

	Properties of PARP inhibitors						
	Olaparib	Veliparib	Talazoparib	Niraparib	Rucaparib	[^18^F]PARPi	PARPi-FL
MW	434.5	244.3	380.8	320.4	323.4	487.5	640.5
PARP1 IC_50_	5 nM^b^	1.2 nM^a^	0.56 nM^a^	3.8 nM^b^	0.65 nM^a^	2.3 nM^b^	12.2 nM^b^
PARP2 IC_50_	1 nM^b^	0.41 nM^a^	0.15 nM^a^	2.1 nM^b^	0.08 nM^a^	0.21 nM^a^	0.31 nM^a^
Trapping^b^	++	+	++++	+++	++	ND	ND
Trials	106	88	20	15	10	0	1
Phase 3	11	6	1	3	1	0	0

^a^ See Supplementary Fig. 2

^b^ Unpublished data^13,18,26,29,30^

### SCLC PDX models show high PARP1 expression levels

**Fig. 2:**
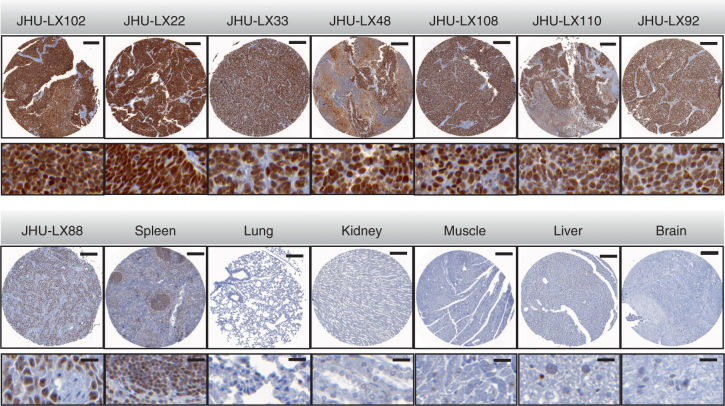
PARP expression in PDX lines. PARP1 expression was assessed via immunohistochemistry staining on tissue microarrays (TMAs) in eight PDX lines which were grown as xenografts in NSG mice and five normal control tissues (spleen, lung, kidney, muscle, liver, and brain). Scale bar represents 200 µm (whole core) and 20 µm (inset). All PDX lines show high PARP1 expression. All PDX lines are SCLC, except JHU-LX88, which is squamous cell lung cancer. These tumors showed lower PARP1 expression than all seven SCLC PDX lines and also showed lower expression than spleen, which has a naturally high PARP1 expression^18^

In order to better understand the possible benefit of PARP targeted therapy and imaging for SCLC, we investigated PARP1 expression in 8 different PDX lines and tissues from 6 different mouse organs (Fig. 2). Using PARP1 specific antibody staining on tissue microarrays, we found that all SCLC PDX exhibited elevated PARP1 expression compared to lung, kidney, muscle, liver and brain. All 7 PDX SCLC lines exhibited greater than 60% PARP1-positive area (between 64 ± 6% PARP1-positive area for JHU-LX92 and 86 ± 3% PARP1-positive area for JHU-LX102), while all organs, except for spleen, exhibited lower than 5% PARP1-positive area (Supplementary Fig. 4), emphasizing the quantitative difference in PARP1 density for binding of PARP1 targeted agents. Spleen showed higher PARP1 expression than the other organs, with a 26 ± 1% PARP1-positive area. The lowest PARP1 expression was seen in lung squamous cell PDX line, JHU-LX88, with 18 ± 6% PARP1-positive area. JHU-LX22 and JHU-LX48 (subsequently used for in vitro and in vivo studies), expression was 85 ± 4% and 78 ± 6% PARP1-positive area, respectively.

### PARPi-FL is capable of imaging target engagement in vitro

**Fig. 3:**
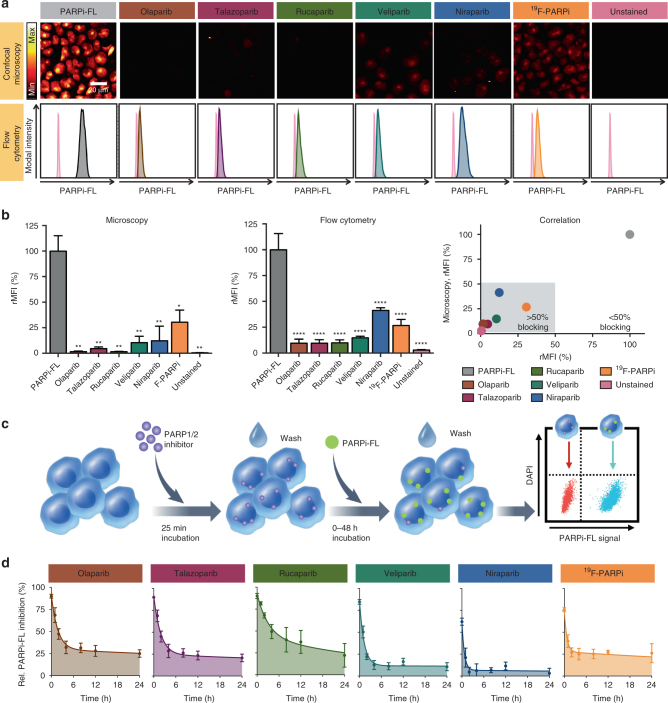
PARPi-FL blocking and in vitro kinetics of different PARP inhibitors. **a** JHU-LX22 SCLC cells were treated with 6 different PARP inhibitors, including 5 clinical therapeutic inhibitors and a cold, non-radioactive analog of the PARP PET tracer, [^18^F]PARPi. Cells were then treated with fluorescent imaging agent PARPi-FL and either imaged via confocal microscopy or measured via flow cytometry. Microscopy (top) and flow cytometry (bottom) showing reduced PARPi-FL uptake in all cells treated with PARP inhibitors. **b** Quantification of PARPi-FL uptake with microscopy (left), flow cytometry (middle) and correlation between the two (right). **c** JHU-LX22 cells were incubated with one of 5 PARP inhibitors (olaparib, talazoparib, rucaparib, veliparib, niraparib, and ^19^F-PARPi) at a concentration of 0.2 µM for 25 min at 37 °C, and washed. After a post-incubation time of 0–48 h, cells were incubated with 0.2 µM PARPi-FL for 15 min at 37 °C, followed by 10 min with medium to allow unbound compound to diffuse out of the cells. Then, cells were trypsinized, subjected to flow cytometry and analyzed for the relative PARPi-FL fluorescence signal. Cells that had not been exposed to a PARP inhibitor served as control and were defined as 0% relative PARP inhibition. **d** Decay curves were calculated with Prism using two phase decay least squares regression. Non-parametric Student’s *t*-test was used to calculate the statistics. *P < 0.05, **P < 0.01, ***P < 0.001, ****P < 0.001. Error bars represent the SD of three independent experiments with three parallels each

In order to determine whether we can use PARP1 imaging agents to probe the characteristics of therapeutic PARP inhibitors, we first assessed whether the fluorescent PARP imaging agent, PARPi-FL, is capable of detecting target engagement of a PARP inhibitor panel in vitro. We tested olaparib, talazoparib, rucaparib, veliparib and niraparib, as well as ^19^F-PARPi. Our goal was to quantify the degree to which PARPi-FL uptake was reduced by the pretreatment of JHU-LX22 with PARP inhibitors, denoting how quantitatively the targeted small molecules occupied the PARP1/2 active site pockets, and, consequently, how efficiently they blocked available binding sites for PARPi-FL. We found that all inhibitors were capable of blocking PARPi-FL uptake, as measured both by confocal microscopy and flow cytometry (Fig. 3a). Quantitation of the blocking showed that the most complete target engagement was observed for olaparib, talazoparib and rucaparib (Fig. 3b). Veliparib, niraparib and ^19^F-PARPi allowed for slightly more binding of PARPi-FL, but still blocked more than 50% of the PARPi-FL uptake (Fig. 3b). A correlation analysis showed that the results from flow cytometry and confocal microscopy were in agreement with each other (Fig. 3b).

### Imaging of PARP inhibitor binding kinetics in vitro

Next, we set out to determine in vitro binding kinetics of the different inhibitors, and to temporally resolve target engagement for the individual drugs. Therefore, we measured PARPi-FL uptake at different time points after PARP inhibitor administration (0–48 h) using flow cytometry (Fig. 3c). This yields an inverse measure of the proportion of binding sites still occupied by the PARP therapeutic. We calculated the target engagement of the PARP inhibitors by fitting a two phase decay curve to the PARPi-FL binding curve. Olaparib, talazoparib, rucaparib and ^19^F-PARPi showed the longest retention on PARP1/2 binding sites, whereas veliparib and niraparib showed faster release kinetics (Fig. 3d). While internally consistent and reproducible, it should be noted that the estimated half-lives for retention using this in vitro methodology are a function of the intensity of the washing procedure, where more intense washing or greater washing medium volumes will shorten the resulting residence time of the drug and also affect the amount of residual inhibition at 48 h. This resembles the in vivo situation, where drugs can experience a seemingly lower residence time when having been deposited in a highly perfused tissue. Recovery to full binding of PARPi-FL could be accelerated by repeated washing (Supplementary Fig. 6) or dilution of the PARP inhibitor concentration (Supplementary Fig. 6). Importantly, the relationship between slower and faster release of drug from the binding sites was consistent across all experimental settings and corroborated our findings.

We also showed that our imaging data reflects target engagement, and not differences in affinity, since the small molecule that first bound to PARP1/2 was dominant at the target site (Supplementary Fig. 8). Changes toward equilibrium binding were only observed at longer incubation times (1–2 h), indicating a slow k_off_ rate. The power and specificity of our approach was further supported by the fact that iniparib, once thought to be a PARP inhibitor^16,17^, failed to reduce PARPi-FL uptake.

We then measured cell viability to determine whether PARPi-FL and ^19^F-PARPi displayed toxicities comparable to their therapeutic counterparts. PARPi-FL and ^19^F-PARPi showed very similar effects on JHU-LX22 cell viability as olaparib. Cells were most sensitive to talazoparib. Veliparib and niraparib, which had shorter in vitro on-target half-lives than olaparib, talazoparib and rucaparib, also showed much less cell killing (Supplementary Fig. 9).

### In vivo [^18^F]PARPi imaging of SCLC PDX models

**Fig. 4:**
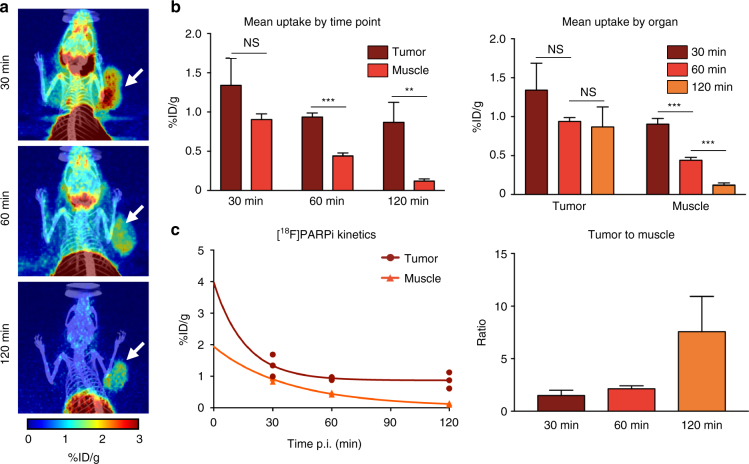
In vivo kinetics of [^18^F]PARPi. JHU-LX48 SCLC PDX bearing mice (*n* = 3/group, 3 group, 9 mice total) were injected (i.v.) with 200–300 µCi of [^18^F]PARPi and imaged via PET/CT at 30, 60, and 120 min post injection. **a** Representative MIPs from a single mouse at all 3 time points. **b** Quantification of PET images for tumor and muscle grouped by time point (left) and grouped by organ (right). **c** Two phase decay curve showing wash out of the tracer from the muscle as the activity in the tumor site remains relatively constant after 60 min (left) along with resulting tumor to muscle ratios (right). Non-parametric Student’s *t*-test was used to calculate the statistics. **P < 0.01, ***P < 0.001. Error bars represent the SD

In order to map the pharmacokinetics of [^18^F]PARPi in the JHU-LX48 PDX model, and to identify the time point that provides ideal tumor/background ratios, we injected tumor bearing mice with [^18^F]PARPi and imaged the mice at 30, 60, and 120 min post injection (Fig. 4a). At 30 min, the tumor was visible above the background signal of muscle (1.34 ± 0.28 %ID/g and 0.90 ± 0.06 %ID/g, respectively), but the differences were not statistically significant (*n* = 3, *P* = 0.1016; Fig. 4b). However, at the 60 min time point, the muscle uptake had begun to clear and activity had fallen sharply to 0.44 ± 0.03 %ID/g, while the tumor signal was retained much more (0.94 ± 0.04 %ID/g), yielding *P* = 0.0002 (Fig. 4b). This trend continued to the 120 min time point, with uptake at 0.12 ± 0.02 %ID/g for the muscle, while tumor uptake remained constant at 0.87 ± 0.21 %ID/g (Fig. 4b). The PARP1/2 imaging agent [^18^F]PARPi is therefore well suited to image in PARP1-expressing tumors, quickly clearing from non-target tissue while binding strongly to tumor over the course of 2 h resulting in a tumor/muscle ratio of 7.6 ± 2.7 in JHU-LX48 bearing animals (*P* = 0.0072; Fig. 4c). We also conducted a biodistribution study and found differences in organ uptake compared to other immunocompromised mouse models used in previous studies^18^ (Supplementary Fig. 5).

### [^18^F]PARPi uptake is similar across tumor sizes

**Fig. 5:**
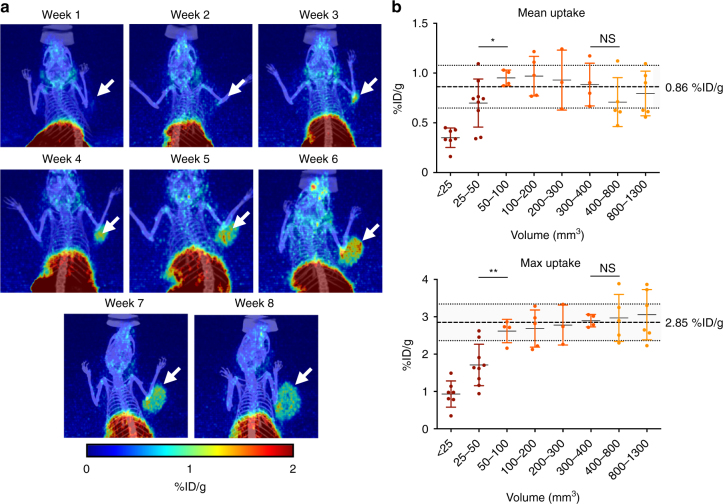
Tumor size to [^18^F]PARPi uptake correlation. JHU-LX48 SCLC PDX bearing mice (*n* = 6) were injected (i.v.) with 200–300 µCi of [^18^F]PARPi and imaged. PET/CT imaging was performed once a week over 8 consecutive weeks starting one week after xenografting. **a** Representative MIPs from the same mouse over 8 weeks. **b** Quantification (*n* = 6) of mean uptake (top) and max uptake (bottom) of the PET images grouped by tumor size. Mean uptake was calculated to be 0.86 %ID/g across a large range, from 50–1300 mm^3^, of tumor sizes as quantified from CT VIOs. Small tumor sizes (<50 mm^3^) are likely affected by partial volume effect^19^. The figure includes the standard deviation as indicated by the shaded area. Non-parametric Student’s *t*-test was used to calculate the statistics. *P < 0.05; **P < 0.01. Error bars represent the SD

In order to obtain quantitative data on how tumor size correlates with tracer uptake, we performed repeated [^18^F]PARPi imaging studies in a cohort of JHU-LX48 PDX mice over the course of 8 weeks (*n* = 6; Fig. 5a). We evaluated the quantitative performance of the imaging probe over tumor volumes ranging from <25 mm^3^ to >1000 mm^3^. Once tumors reached a size greater than 50 mm^3^, tumor size had no statistically significant effect on the mean [^18^F]PARPi signal. Mean signal remained steady at 0.86 ± 0.22 %ID/g for sizes ranging 50 mm^3^ to 1300 mm^3^ with the highest single value at 1.24 %ID/g and the lowest single value at 0.47 %ID/g (Fig. 5b). Separating the data into groups organized by tumor size, we observed that at very low tumor volumes (<50 mm^3^) uptake values appear lower, presumably because of the partial volume effect^19^ and the associated detection limit of the small animal PET/CT used for these experiments. The highest mean uptake values (0.97 ± 0.18 %ID/g) were observed for tumor sizes between 100 and 200 mm^3^. With larger tumor sizes, mean signal decreased slightly until reaching a minimum of 0.71 ± 0.22 %ID/g for tumor sizes between 400 and 800 mm^3^, presumably due to an increasing fraction of necrotic tissue in larger tumors. Necrotic tissue does not retain [^18^F]PARPi, and therefore reduces the mean uptake. This was confirmed by the maximum uptake values (Fig. 5b). Organized into groups, the maximum uptake showed a continuous increase as tumor size increased in this range, from 2.62 ± 0.27 %ID/g for 50–100 mm^3^ to 3.06 ± 0.61 %ID/g for 800–1300 mm^3^ (Fig. 5b). Maximum uptake across tumors sizes 50–1300 mm^3^ was 2.85 ± 0.49 %ID/g.

### Ex vivo autoradiography measurements of target engagement

**Fig. 6:**
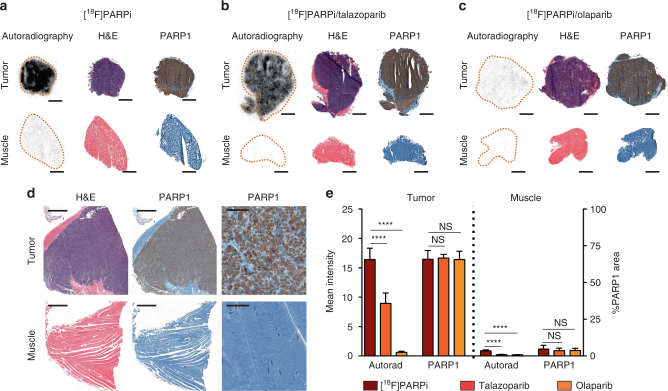
Autoradiography. JHU-LX48 SCLC PDX bearing mice (*n* = 3/group, 3 groups, 9 mice total) were injected either with 200–300 μCi of [^18^F]PARPi (i.v.) alone, or with a previous injection of talazoparib (0.3 mg/kg) or olaparib (50 mg/kg) 30 min prior to [^18^F]PARPi. Tumor (top) and muscle (bottom) were collected 2 h post [^18^F]PARPi injection, sectioned and either exposed overnight (autoradiography), stained with H&E or stained for PARP1. Example images are shown for **a** [^18^F]PARPi alone, **b** [^18^F]PARPi with a prior injection of talazoparib and **c** [^18^F]PARPi with a prior injection of olaparib. **d** Higher magnification of PARP1 IHC of a tumor and muscle sample. **e** Quantification of autoradiography signal and PARP1 expression. Non-parametric Student’s *t*-test was used to calculate the statistics. ****P < 0.0001. Displayed data represent means with SD from three sections of three tumors/group

In the preclinical setting, olaparib is administered at dosing levels of 50 mg/kg, whereas talazoparib is used at much lower doses (e.g., 0.3 mg/kg)^20^. These trends are also reflected in the clinical setting (protocols: NCT02032823, NCT02184195, NCT01945775). Target engagement studies using olaparib and talazoparib showed that both molecules were capable of reducing [^18^F]PARPi signal in tumor and muscle tissue when administered 30 min prior to the tracer (Fig. 6a–c). Using PARP1 IHC staining, we could show comparably high PARP1 expression in tumors of all groups, while expression in muscle was very low, confirming validity of the autoradiography findings (Fig. 6d). Target engagement was not equivalent for olaparib and talazoparib at their respective therapeutically active doses. Autoradiography demonstrated that doses of olaparib were capable of completely blocking tumor uptake (96% of [^18^F]PARPi uptake blocked), while talazoparib was only capable of blocking tumor uptake by 45% (Fig. 6e). A significant reduction of [^18^F]PARPi was also observed in muscle tissue, despite the low PARP1 expression.

### Target engagement differs for different PARP therapeutics

**Fig. 7:**
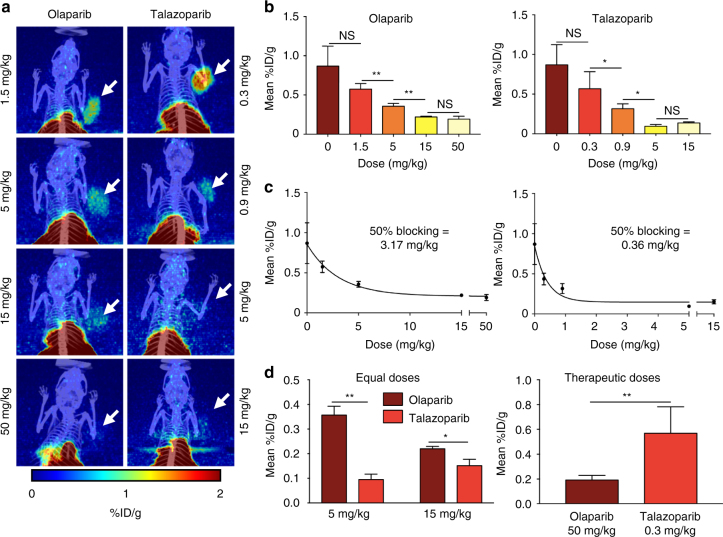
Variable dose blocking. JHU-LX48 SCLC PDX bearing mice (*n* = 3/group, 9 groups, 27 total) were injected (i.v.) first with olaparib (0–50 mg/kg) or talazoparib (0–15 mg/kg). Mice were injected (i.v.) with 200–300 μCi of [^18^F]PARPi 30 min later, and imaged via PET/CT 2 h later. **a** Representative MIPs from 4 different doses each, for olaparib (left) and talazoparib (right). **b** Quantification of PET images for tumor uptake at each of 4 different doses of olaparib (left) and talazoparib (right), compared to [^18^F]PARPi only. **c** Curve fitting analysis for olaparib (left) and talazoparib (right) showing calculated 50% blocking doses. **d** Comparison of olaparib and talazoparib at equal doses (left) and at therapeutic doses (right). Non-parametric Student’s *t*-test was used to calculate the statistics. *P < 0.05; **P < 0.01. Error bars represent the SD

Since we saw that talazoparib, at the therapeutic doses used preclinically^20^, does not exhibit complete target engagement we next aimed to study different doses of olaparib and talazoparib and to correlate these doses to the level of target engagement as shown by [^18^F]PARPi blocking (Fig. 7). A positive control cohort (receiving vehicle control instead of PARP inhibitor) showed a mean [^18^F]PARPi uptake of 0.87 ± 0.21 %ID/g.

The results for olaparib showed that the therapeutic dose level is much higher than required to achieve near complete target engagement. Doses of 50 mg/kg showed 78% blocking with a mean uptake of 0.19 ± 0.03 %ID/g, and 15 mg/kg resulted in similar 75% blocking with a mean uptake of 0.22 ± 0.01 %ID/g (*P* > 0.25; Fig. 7b). When we reduced the dose to 5 mg/kg we saw a significant reduction to 59% target engagement (0.36 ± 0.03 %ID/g, *P* < 0.005), compared to the control cohort. At 1.5 mg/kg target engagement was further reduced to 34% (0.57 ± 0.06 %ID/g, *P* < 0.001). This value is not statistically different from the control values (*P* > 0.1). We fit a single-phase decay curve (*R*^2^ = 0.8629) to this data (Fig. 7c, left) to calculate the 50% blocking dose to be 3.17 mg/kg.

For talazoparib, the therapeutic dose was found to be insufficient to achieve complete target engagement. Doses of 0.3 mg/kg resulted in 50% target engagement (0.44 ± 0.06 %ID/g [^18^F]PARPi in tumor tissue). An increase of the administered dose to 0.9 mg/kg further increased the target engagement to 64% (0.32 ± 0.05 %ID/g, *P* < 0.05). Increase in dose to 5 mg/kg resulted in 89% target engagement (0.10 ± 0.02 %ID/g, *P* < 0.05), while 15 mg/kg showed no further increase in target engagement (84%, 0.14 ± 0.01%ID/g, *P* > 0.05) (Fig. 7b). A single phase decay curve (*R*^2^ = 0.8462) (Fig. 7c, right) was used to calculate the 50% blocking dose to be 0.36 mg/kg.

Interestingly, while target engagement was more complete for olaparib when using doses used in preclinical therapeutic studies (78 and 50% for olaparib and talazoparib, respectively; Fig. 7d), talazoparib achieved a more quantitative target engagement at similar doses (59% and 89% for olaparib and talazoparib at 5 mg/kg, respectively, 75% and 84% for olaparib and talazoparib at 15 mg/kg, respectively; Fig. 7d).

### [^18^F]PARPi detects differences in kinetic properties in vivo

**Fig. 8:**
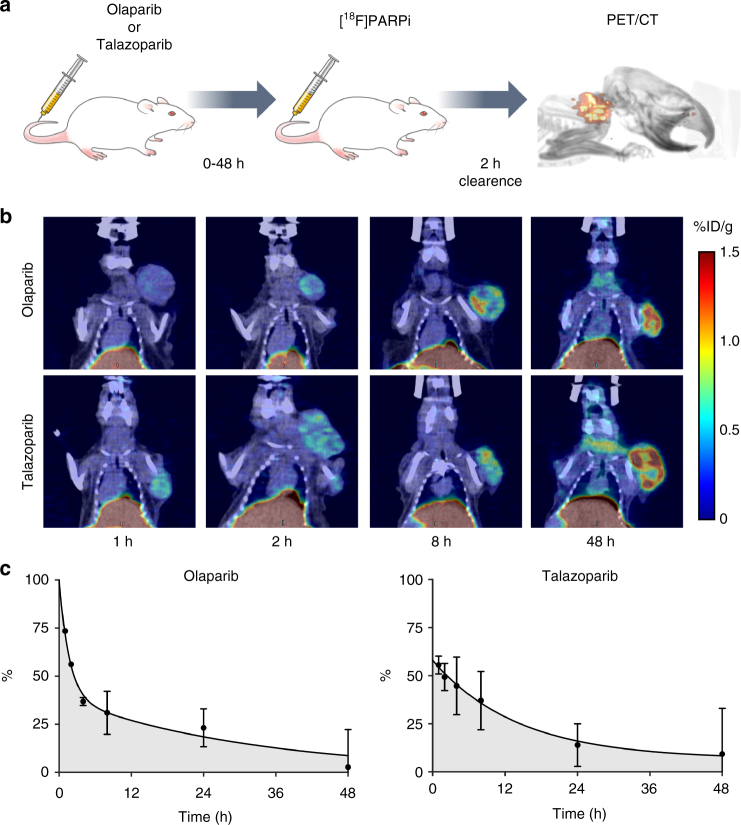
In vivo kinetics of different PARP inhibitors. **a** JHU-LX48 SCLC PDX bearing mice (*n* = 3/group, 15 groups, 45 mice total) were injected (i.v.) first with olaparib (50 mg/kg) or talazoparib (0.3 mg/kg). Mice were injected (i.v.) with 200–300 µCi of [^18^F]PARPi 1, 2, 4, 8, 24, or 48 h later, and imaged via PET/CT 2 h post [^18^F]PARPi injection. **b** Representative coronal slices from 4 different time points after olaparib (left) and talazoparib (right) administration. **c** Quantification of PET images for tumor uptake at each of 6 different time points represented as a percentage of blocked signal. Error bars represent the SD

The dosing studies showed a significant difference between olaparib and talazoparib in their ability to block [^18^F]PARPi signal in the tumor at a single defined time point. An important factor in measuring the deposited effective doses, however, is how long the inhibitor occupies the binding site. Therefore we sought to determine the residence time of olaparib and talazoparib in vivo. We conducted experiments to measure the on-target residence time using [^18^F]PARPi by varying the amount of time (1 to 48 h) between administration of the drug and administration of the tracer (Fig. 8a). For olaparib, we found that, at 50 mg/kg, an initial scan showed 74% target engagement (0.24 ± 0.00 %ID/g) at 60 min between injection of olaparib and [^18^F]PARPi. Mice with longer time intervals between injection of drug and tracer showed gradual increase in [^18^F]PARPi signal (Fig. 8b, top) until, at 48 h, we measured tumor uptake to be 0.88 ± 0.15 %ID/g, which was not statistically different from the control cohort of mice which did not receive olaparib. We fit a two phase decay curve (R^2^ = 0.8548) to the target engagement data and used this to calculate a weighted half-life of 9.4 h (Fig. 8c, left). This number represents the amount of time it took for half of the [^18^F]PARPi signal in the tumor to return after a (therapeutic) dose of 50 mg/kg olaparib. We also used this curve to calculate an area under the curve, which was 1078 %h for olaparib. This number is a measure for the target engagement of a single dose of olaparib (50 mg/kg) over 48 h. Complete 100% target engagement over 48 h would result in an integral of 4800 %h.

We repeated this procedure for talazoparib and found that, at 0.3 mg/kg, the 60 min time point showed 0.40 ± 0.02 %ID/g with 56% target engagement. Again, we found a gradual increase in [^18^F]PARPi signal as we increased the interval between drug and tracer (Fig. 8b, right). At 48 h, we measured 0.82 ± 0.18 %ID/g, which was not statistically different from the control cohort of mice which did not receive talazoparib. We fit a two phase decay curve (R^2^ = 0.6328) to the target engagement data and used this to calculate a weighted half-life of 9.8 h (Fig. 8c, right). This number represents the amount of time it took for half of the [^18^F]PARPi signal in the tumor to return after a dose of 0.3 mg/kg talazoparib. We also used this curve to calculate an area under the curve, which was 1021 %h. This number represents the total amount of [^18^F]PARPi signal blocking a single dose of 0.3 mg/kg talazoparib provides over the course of 48 h.

Hence, we found comparable tumor residence times for olaparib and talazoparib, despite differences in the applied dose, differences in the initial target engagement and reported differences in affinity and trapping potential (Table 2).

**Table 2:** Summary of in vivo kinetics data of olaparib and talazoparib

	Initial blocking (*t* = 1 h)	*t*_1/2_ (weighted)	AUC
Olaparib	74%	9.4 h	1078 %h
Talazoparib	56%	9.8 h	1021 %h
Ratio_T:O_	0.76	1.04	0.95

In vivo kinetics measurements

## Discussion

In this study, we demonstrated the feasibility of quantitative in vivo target engagement of a family of PARP inhibitors in SCLC PDX models using a molecularly targeted PET imaging agent. An imaging agent like this could have important implications for PARP inhibitor treatment planning and monitoring in the clinic.

Currently, there are several, molecularly targeted PARP therapeutics which are being tested in the clinic. Despite the universal importance of PARP for DNA repair, response to PARP inhibitor treatment can vary dramatically between patients. This observation has prompted a number of investigations aimed at determining biomarkers for PARP inhibitor treatment. These studies have identified deficiencies in other DNA repair pathways, such as BRCA1/2^21,22^ or expression of SLFN11^23,24^, as important factors that increase tumor cell sensitivity to PARP inhibition. We expect that within genetically similar patient cohorts, however, sufficient target expression, drug delivery and target engagement are likely to be foundational pillars for effective PARP inhibitor treatment. Invasive tumor biopsy has some utility in assessing target expression, but a small core sample of a single lesion will not capture tumor heterogeneity of expression and cannot provide insight into the dynamics of target engagement and drug delivery. It is currently not possible to measure if PARP inhibitor intratumoral delivery, target engagement and duration of target suppression are sufficient to elicit a response. Such data could provide a faster feedback and uniquely valuable information in planning and adapting treatment regimens.

The need for such approaches has recently been underlined by the failure of iniparib. Iniparib was believed to be a PARP inhibitor^16,17^ but failed in a phase III clinical trial when it was realized that iniparib does not actually inhibit PARP. The inability of iniparib to block binding of PARPi-FL was immediately evident using our assay, and could have informed decisions early in its development, even prior to clinical testing.

Interestingly, our in vitro data suggests that different PARP inhibitors have varying residence times in tumor cells. This variable could contribute to the diverse treatment efficacies that have been observed^25^. Intracellular residence times could represent an additional measurement in evaluating PARP inhibitor efficacy in addition to IC_50_s and PARP trapping^14^.

We have shown that our fluorescently labeled and ^18^F-labeled PARP imaging probes are capable of quantifying PARP inhibitor target engagement. Both showed high specificity for PARP1 and PARP2, key members of the PARP enzyme family. Our study, which measured enzymatic activity at 100 nM of inhibitor, differs methodologically from a previous study which utilized differential scanning fluorimetry (DSF) to produce cross family reactivity profiles^7^, but yielded generally similar binding profiles for olaparib, veliparib, and rucaparib with respect to PARP1–3. Both imaging agents showed selective inhibition for PARP1/2 while showing less inhibition for PARP3. This is somewhat surprising given that both imaging agents contain a 1-(2 H)-phthalazinone, pharmacophore similar to olaparib, which shows inhibition of PARP3. Because of their exceptional selectivity and strong binding profile, our imaging agents were indeed able to quantitatively determine target engagement across the entire class of clinically active PARP inhibitors. We have shown that PARPi-FL can image target engagement for five clinical inhibitors currently either in phase III trials or FDA approved. We have also shown that [^18^F]PARPi can be used to detect target engagement for olaparib and talazoparib, both therapeutic inhibitors which are of special interest for SCLC^6,23^. Importantly, the imaging agents were capable of determining both partial target engagement as well as complete target engagement. Identification of incomplete target engagement with a radiolabeled PARP imaging agent in a clinical treatment setting could potentially allow for adjustments of the PARP inhibitor dose to avoid underdosing and, therefore, increase the likelihood of therapeutic success. This type of imaging could be performed before, after and during a treatment cycle, because, for [^18^F]PARPi (0.97 Ci/μmol), only about 10 μg of material needs be injected to obtain good signal/noise ratios in a PET/CT scanner (10 mCi/patient) whereas therapeutics are administered in much higher quantities. Currently, human dosing for olaparib is 600 mg per day (300 mg twice daily, NCT02032823, NCT02184195), and even talazoparib is being tested at 1 mg per day (NCT01945775). This indicates that an injection of [^18^F]PARPi is a tracer dose, 100 times less than the daily dose of talazoparib and 60,000 times less than that of olaparib.

Characterization of olaparib and talazoparib using [^18^F]PARPi PET quantitatively illustrated the differences between the two PARP inhibitors. We found that doses previously used to provide therapeutic effects in the preclinical setting (50 mg/kg for olaparib, 0.3 mg/kg for talazoparib)^26,27^ are higher than necessary for olaparib and lower than necessary for talazoparib to provide complete saturation of the available binding sites at the tumor. This might lend credence to the preclinical evidence that the two drugs offer efficacy that varies with potency of different mechanisms (PARP inhibition vs. PARP trapping)^28^. Our kinetic studies showed that a slightly faster dissociation for olaparib resulted in a very similar overall effective dose as measured by integrating the occupation of the binding sites over the course of 48 h, and that this value (Fig. 8c) might be a valuable pharmacodynamic parameter.

In summary, the molecular imaging oriented approach to drug characterization and target engagement measurement presented here provides a robust and adaptable method for answering important questions regarding the interaction of a targeted drug with its intended binding site in a quantifiable manner in vitro and in vivo. Imaging techniques like this have the potential not only to revolutionize the way that drugs are prepared but also used in the clinic, and could pave the way for more robust and successful patient selection and treatment monitoring. More specifically, our PET imaging agent [^18^F]PARPi might be used to quantify target engagement of phase III and FDA approved PARP1 inhibitors in vivo without the need of creating individual companion imaging agents for each drug. This non-invasive, whole body approach could potentially preclude the need for multiple temporally and spatially separated biopsies, and would allow quantification of target engagement for each lesion in an individual. [^18^F]PARPi PET may provide a robust tool for treatment and patient selection with profound clinical-translational implications.

## Methods

### General

High performance liquid chromatography (HPLC) purification and analysis was performed on a Shimadzu UFLC HPLC system equipped with a DGU-20A degasser, a SPD-M20A UV detector, a LC-20AB pump system, and a CBM-20A communication BUS module. A LabLogic Scan-RAM radio-TLC/HPLC-detector was used for the radioactive signal. HPLC solvents (Buffer A: 0.1% TFA in water, Buffer B: 0.1% TFA in MeCN) were filtered before use. HPLC purification was performed on a semi-prep reversed phase Phenomenex Gemini column (C6-Phenyl, 5 μm, 10 mm, and 250 mm) while analysis was performed on an analytical reversed phase Phenomenex Gemini column (C18, 5 μm, 4.6 mm, and 250 mm). Purification was performed with Method A (flowrate: 5 mL/min; isocratic: 0–45 min 30% B). Analysis was performed with Method B (flowrate: 1.5 mL/min; gradient: 0–14 min 5–100% B; 14–18 min 100% B; 18–18.5 min 100%-5% B; 18.5–22 min 5% B). All PET imaging experiments were conducted on a microPET INVEON camera equipped with a CT scanner (Siemens, Knoxville, TN). Digital phosphor autoradiography was obtained using a Typhoon FLA 7000 laser scanner from GE Healthcare (Port Washington, NY).

### Chemicals

Commercially available compounds were used without further purification unless otherwise stated. 4,7,13,16,21,24-Hexaoxa-1,10-diazabicyclo[8.8.8]hexacosane (K_222_), extra dry dimethyl sulfoxide (DMSO) over molecular sieves, Ethyl 4-nitrobenzoate, and 4-Fluorobenzoic acid were purchased from Sigma-Aldrich (St. Louis, MO). Water (>18.2 MΩcm-1 at 25 °C) was obtained from an Alpha-Q Ultrapure water system from Millipore (Bedford, MA). Commercially available PARP inhibitors were purchased through SelleckChem. PARP inhibitors were kept as 10 mM stock solutions in DMSO and diluted to the final working concentration for the respective in vitro experiment with full medium.

### PARPi-FL synthesis

The green fluorescent dye BODIPY-FL NHS-ester (Invitrogen, Carlsbad, CA) was conjugated to 4-(4-fluoro-3-(piperazine-1-carbonyl)benzyl)phthalazin-1(2H)-one and purified by preparative HPLC (Waters’ XTerra C-18 5 μm column, 7 mL/min, 5 to 95% of acetonitrile in 15 min) to afford PARPi-FL in 70–79% yield as a red solid. Analytical HPLC analysis (Waters’ Atlantis T3 C18 5 μm 4.6 × 250 mm column) showed high purity (>97%) of the imaging agent. The identity of PARPi-FL was confirmed using ESI-MS (MS(+) *m/z* = 663.4 [M + Na]+).

### ^19^F-PARPi synthesis

4-(4-fluoro-3-(piperazine-1-carbonyl)benzyl)phthalazin- 1(2H)-one was dissolved in MeCN and 4-fluorobenzoic acid was added followed by N,N,N’,N’-Tetramethyl-O-(1H-benzotriazol-1-yl)uronium hexafluorophosphate (HBTU) and Et_3_N. The reaction mixture was stirred for 5 min and purified by preparative HPLC (Waters’ XTerra C-18 5 μm column, 7 mL/min, 5 to 95% of acetonitrile in 15 min) to produce the compound in 40–50% yield as a white solid. Analytical HPLC analysis (Waters’ Atlantis T3 C18 5 μm 4.6 × 250 mm column) showed high purity (>97%) and the identity was confirmed using ESI-MS (MS(+)* m/z* = 511.2 [M + Na]+).

### Radiosynthesis

[^18^F]PARPi was synthesized using an optimized labeling procedure. No-carrier-added (n.c.a.) [^18^F]fluoride was obtained via the ^18^O(p,n)^18^F nuclear reaction of 16.5-MeV protons in an GE Healthcare PETTrace 800 using enriched ^18^O-water. QMA light ion-exchange cartridges and C-18 light Sep-Pak cartridges were obtained from Waters (Milford, MA). A QMA cartridge containing cyclotron-produced [^18^F] fluoride ion (50 mCi, 1.85 GBq) was eluted with a solution containing 9 mg Kryptofix [2.2.2] (4,7,13,16,21,24-hexaoxa-1,10-diazabicyclo[8.8.8]hexacosane), 0.08 mL 0.15 M K2CO3 and 1.92 mL MeCN into a 5 mL reaction vial. Solvents were removed azeotropically at 120 °C under N_2_. Afterwards, 500 µg of ethyl 4-nitrobenzoate in 100 µL of dry DMSO was added and the mixture was heated to 150 °C for 15 min. 50 µL of 1 M NaOH was then added followed by 50 µL of 1 M HCl. Then, 2 mg of 4-(4-fluoro-3-(piperazine-1-carbonyl)benzyl)phthalazin-1(2H)-one in 100 µL of dry DMSO was added followed by 10 mg of HBTU dissolved in 100 µL of DMSO and 20 µL of Et_3_N. 400 µL of MeCN and 1 mL H_2_O were then added to the mixture and the product was purified by HPLC (Method A, *t*_R_ = 32 min) yielding (n.d.c) 38.4 ± 2.5% with a SA of 0.97 Ci/µmol.

### PARP family binding assay

Assay was performed by BPS Bioscience, San Diego, CA. 100 µM DMSO solutions of each inhibitor were provided, and the assay was conducted after dilution to 100, 50, or 10 nM. The measurements for each enzyme combined with each inhibitor was performed in triplicate according the BPS assay kit protocols. Luminescence was measured using a BioTek Synergy 2 microplate reader. Data was reported by BPS as % Enzyme Activity. Triplicates were combined to get a mean ± SD, and these were grouped and plotted onto a heatmap using Prism 7.

### PARP1 and PARP2 IC_50_s

Assays were performed by BPS Bioscience, San Diego, CA. 100 µM DMSO solutions of ^19^F-PARPi, PARPi-FL, and rucaparib were provided to BPS and other inhibitors were provided by BPS. Each measurement was performed in triplicate according the BPS assay kit protocols. Luminescence was measured using a BioTek Synergy 2 microplate reader. Data was reported by BPS a`s % enzyme activity curve from 0.03 nM or 0.1 nM to 1000 nM.

### PARylation inhibition assay

LX22 cells (5 × 10^6^ cells/sample) were treated with 0.1 µM or 1 µM PARPi-FL, [^18^F]PARPi or olaparib in medium for 30 min at 37 °C or left untreated. Whole cell lysates were prepared by incubating cells with RIPA Buffer with Triton X (BP-140, Boston BioProducts) containing Protease Inhibitor (Complete EASYpacks, Sigma-Aldrich) for 30 min on ice, followed by 30 min centrifugation at 10,000 × *g* for 30 min at 4 °C. Protein concentration of lysates was determined using a Bicinchoninic acid (BCA) assay kit (#23225, Pierce) and following the manufacturers instructions. SDS gel electrophoresis and immunoblotting were carried out following standard procedures. Signal detection was carried out using chemiluminescent substrate (#34077, Thermo Scientific). Densitometric analysis of western blots was carried out using ImageJ (NIH). To detect PAR, we used a rabbit polyclonal anti-PAR polymer antibody (1:1000 dilution, #4336-BPC-100, Trevigen) followed by a goat anti-rabbit IgG-HRP secondary antibody (1:10,000 dilution, sc-2004, SantaCruz). B-actin was used as loading control (1:1000 dilution, A3854, Sigma-Aldrich) and was stained after stripping the blot for 30 min at room temperature using stripping buffer (Amresco).

### Tissue microarray and PARP1 immunohistochemistry

PARP1 Immunohistochemistry staining was carried out on tissue microarrays of PDX models of SCLC, which were assembled from standard formalin-fixed paraffin embedded (FFPE) PDX tissue blocks. Briefly, after antigen retrieval and blocking, sections were incubated with anti-PARP1 primary antibody for 5 h (1:10,000; 0.02 µg/mL; 0.2 µg/mL; sc-7150, Santa Cruz Biotechnology; The antibody used for PARP1 staining was since discontinued. Santa Cruz now offers the monoclonal anti-PARP1 antibody [sc-8007], which we validated for PARP1 staining at a concentration of 0.4 µg/mL), followed by 1 h with biotinylated goat anti-rabbit IgG (PK6106, Vector Labs). For detection, a DAB detection kit (Ventana Medical Systems) was used according to the manufacturer’s instructions. Sections were counterstained with hematoxylin and cover-slipped with Permount (Fisher Scientific). Staining was carried out using the automated Discovery XT processor (Ventana Medical Systems) at the Molecular Cytology Core Facility at MSK. Tissue microarrays were digitalized using a MIRAX Slide Scanner (3DHISTECH). For PARP1 protein quantification, we calculated the PARP1-positive area for each tissue core using MetaMorph Software (Molecular Devices, Sunnyvale, CA). Thresholding was performed on brown (PARP1) and blue (tissue) areas and the relative PARP1-positive area was calculated by dividing the brown area by the total tissue area. For each PDX line, three cores of three separate animals were available (up to nine total). If more than 50% of the area was necrotic, the core was excluded. For each line, we evaluated at least four different cores.

### Fluorescence microscopy

We conducted confocal laser scanning microscopy to visualize the extent of intracellular blocking of PARPi-FL uptake by different PARPi. Therefore, JHU-LX22 cells were seeded into 8-Well Chamber Slides (Millipore) at a density of 20,000 cells per well and allowed to attach for 24 h at 37 °C. Then, cells were incubated with 0.5 µM olaparib, talazoparib, rucaparib, veliparib, niraparib, ^19^F-PARPi or medium for 25 min, followed by a wash with medium. Then, 0.5 µM PARPi-FL were added to all wells, except the unstained control and incubated for 15 min at 37 °C. The incubation time of PARPi-FL was within the range of linear uptake (Supplementary Fig. 9). PARPi-FL was removed and cells were washed in fresh medium for 10 min. Next, cells were treated with 4% Paraformaldehyde (PFA) for 8 min on ice to ensure fixation. After a final wash with PBS, chambers were removed and slides were mounted with Mowiol mounting medium containing Hoechst 33342 for counterstaining of nuclei. Fluorescence was analyzed using a Leica SP5 upright confocal microscope (Leica, Buffalo Grove, IL) equipped with appropriate laser excitation (Hoechst: 405 nm and PARPi-FL: 488 nm) and matched emission filters. PARPi-FL intensity was determined by measuring the PARPi-FL fluorescence intensity in all nuclei, which were thresholded using Hoechst staining. The measured fluorescence intensities were averaged over 3 fields of view per experimental condition, which contained on average 144 ± 63 nuclei and reported as relative mean fluorescence intensities (rMFI), where PARPi-FL uptake without blocking was defined as 100%.

### Flow cytometry

For all flow cytometry experiments, 200,000 JHU-LX22 cells were seeded into 6-Well plates and allowed to attach for 24 h. After the respective treatment/staining, which is described below for the different experiments, cells were washed in PBS, trypsinized, washed with flow buffer (1% BSA (w/v) in PBS), transferred to 5 mL flow cytometry tubes through the 40 µm cell strainer cap and left on ice until measurement in the flow cytometer (Fortessa II). Raw data were processed in FlowJo software in order to calculate PARPi-FL uptake per cell. Dead cells were excluded by eliminating DAPI positive cells from the analysis (Supplementary Fig. 8). Cell clumps and debris were eliminated using the corresponding gates (forward and side scatter) for the unstained cell population. PARPi-FL fluorescence was imaged in the FITC channel against side scatter (area).

### Target recovery-washing

We compared how the recovery of PARPi-FL binding changed depending on washing regimes. Therefore, JHU-LX22 cells were incubated with one of five PARP inhibitors (olaparib, talazoparib, rucaparib, veliparib, niraparib) at a concentration of 0.2 µM for 25 min at 37 °C and washed. Then, fresh media was added for a post-incubation time of 0, 12, 24, or 48 h. Cells were either left in the same medium for the entire post-incubation time or additional media exchanges were performed after 6, 20, and 40 h. Subsequently, cells were incubated with 0.2 µM PARPi-FL for 15 min at 37 °C, followed by 10 min wash with medium to allow unbound compound to diffuse out of the cells. Then, cells were subjected to flow cytometry as described above.

### Target recovery-dilution

Here, we compared how the recovery of PARPi-FL binding depends on the applied PARPi concentration. JHU-LX22 cells were incubated with one of five PARP inhibitors (olaparib, talazoparib, rucaparib, veliparib, and niraparib) at a concentration of 0.02, 0,1, 0,2, 2, 5, or 12.5 µM for 25 min at 37 °C, washed and supplied with fresh media for the post incubation time of 2 or 24 h. Then, cells were incubated with 0.2 µM PARPi-FL for 15 min at 37 °C, followed by 10 min wash with medium and subjected to flow cytometry as described above.

### PARPi-FL uptake kinetics

The experiment was conducted as described in “Flow Cytometry” in the Methods section. The staining in this experiment consisted of incubation of the JHU-LX22 cells with 0.2 µM PARPi-FL for 1, 2, 5, 10, 20, 30, 45, 60 or 120 min.

### Competition kinetics

To confirm that the measured in vitro kinetics depend on target engagement and did not merely reflect differences in affinity of the different PARP inhibitors, we tested if the release of PARP inhibitors from the cells was faster in presence of PARPi-FL and vice versa. Therefore, we incubated JHU-LX22 cells first with a PARPi (olaparib, talazoparib, rucaparib, veliparib, niraparib, and niraparib) in a concentration of 0.2 µM or medium (control) for 20 min at 37 °C, followed by a brief wash in media and an incubation with 0.2 µM PARPi-FL for 20, 60, or 120 min followed by 10 min wash with medium before cells were subjected to flow cytometry as described above. To show it is more important which molecule bound first instead of which has the higher affinity we also inverted the incubation scheme and incubated the JHU-LX22 cells first with 0.2 µM PARPi-FL for 20 min, followed by 0.2 µM of a PARPi (olaparib, talazoparib, rucaparib, veliparib, niraparib) or medium (control) for 20 min at 37 °C, followed by a 10 min media wash before cells were processed for flow cytometry. Controls (cells incubated with medium instead of a PARP inhibitor) served as reference value and their PARPi-FL signal was defined as 100%.

### Cytotoxicity

Here, we aimed to measure cytotoxic effects of the PARPi (olaparib, talazoparib, rucaparib, veliparib, and niraparib) and imaging compounds (PARPi-FL, ^19^F-PARPi) on JHU-LX22 cells using the Alamar Blue assay (Invitrogen). Cells were plated in 96-well plates (black, clear-bottom, Corning Incorporated) at a density of 5,000 cells/well and allowed them to attach for 24 h. All compounds were prepared as 10 mM stock solutions in DMSO and further diluted in media. Cells were treated with 0, 2, 5, and 10 µM (final solutions containing 1% DMSO) of each compound for 24, 48, 72 and 96 h. Then, media was removed and Alamar Blue reagent was added to each well. Plates were incubated at 37 °C for 4 h under protection from direct light. Fluorescence was read (bottom-read mode) using an excitation wavelength of 570 nm and an emission wavelength of 585 nm on a SpectraMax M5 microplate reader (Molecular Devices, Sunnyvale, CA, USA). The average reading values were analyzed by subtracting the background readings (wells with Alamar Blue but without cells) and compared against the untreated controls of each plate (defined as 100% viability) to determine the percentage of living cells. The experiment was carried out in three biological replicates with four parallels of each compound. The displayed data represent means and standard errors.

### Extent of PARPi-FL blocking by various PARPi

To quantify the extent of blocking of PARPi-FL uptake by different PARPi, we quantified the intracellular PARPi-FL uptake in JHU-LX22 cells after incubation with PARP inhibitors. Therefore, PARPi (olaparib, talazoparib, rucaparib, veliparib, niraparib, and ^19^F-PARPi) were added to wells at a concentration of 0.2 µM for 25 min at 37 °C. After a brief wash with medium, cells were incubated with 0.2 µM PARPi-FL for 15 min at 37 °C and washed for 10 min in medium, followed by the flow cytometry protocol described above.

### In vitro Kinetics

We furthermore determined the duration of PARPi binding to their target site. Therefore, JHU-LX22 cells were incubated with one of five PARP inhibitors (olaparib, talazoparib, rucaparib, veliparib, niraparib, or ^19^F-PARPi) at a concentration of 0.2 µM for 25 min at 37 °C and washed. Then, fresh media was added for a post-incubation time of 0, 1, 2, 4, 8, 12, 24, or 48 h. Subsequently, cells were incubated with 0.2 µM PARPi-FL for 15 min at 37 °C, followed by 10 min wash with medium to allow unbound compound to diffuse out of the cells. Then, cells were subjected to flow cytometry as described above. Cells that have not been exposed to a PARP inhibitor served as control, and were defined as 0% relative PARP inhibition. We determined the duration of binding of the PARPi indirectly by calculating the recovery of PARP-FL binding with increasing times between PARPi and PARPi-FL treatment. Decay curves were calculated with Prism using two phase decay least squares regression.

### Animals

We used 12 nude and 24 NSG mice for biodistribution, and 96 NSG mice for imaging experiments. Female athymic nude CrTac:NCr-Foxn1nu mice at age 6–8 weeks were purchased from Taconic Laboratories (Hudson, NY). Female NSG mice (NOD.Cg-Prkdc^scid^ Il2rg^tm1Wjl^/SzJ; The Jackson Laboratory) were 6 to 8 weeks old at time of PDX injection/implantation. All animal experiments were done in accordance with protocols approved by the Institutional Animal Care and Use Committee (IACUC) of Memorial Sloan Kettering Cancer Center (MSKCC) and followed the National Institutes of Health guidelines for animal welfare.

### SCLC PDX Models

PDX lines were derived at Johns Hopkins University from patients with extensive stage SCLC^31,32^. After PDX model establishment, tumors were serial passaged and expanded into 6-week-old to 8-week-old female NSG mice. To establish experimental cohorts, ~ 300 mg of fresh tumor tissue was dissociated into single cell suspension in 5 mL of RPMI 1640 supplemented with human tumor dissociation enzyme kit in a gentleMACS C tube using an octoMACS automated tissue dissociator (Miltenyi). 5 × 10^6^ viable cells/mouse were injected with a 1:1 mix of Hank’s balanced salt solution and Matrigel basement membrane (Corning) in a final volume of 100 μL subcutaneously into the right shoulder. PDX identity was confirmed by Short Tandem Repeat analysis using the PowerPlex 18 panel (Promega, Madison, WI, USA; DDC Medical is Fairfield, OH, USA). Because of the lines’ inherent varying preferences to grow in vitro/propagate in vivo, we chose LX22 for all in vitro experiments, whereas LX48 was chosen for in vivo experiments. All animal experiments were approved by the Institutional Animal Care and Use Committee (IACUC) of Memorial Sloan Kettering Cancer Center.

### Biodistribution

Healthy nude (*n* = 6/group, 2 groups, 12 total), healthy NSG (*n* = 6/group, 2 groups, 12 total), or tumor bearing NSG (*n* = 6/group, 2 groups, 12 total) mice were injected either with or olaparib as a blocking agent (50 mg/kg) 30 min prior to injections of 200–300 μCi of [^18^F]PARPi in 100–200 μL 10% EtOH in 0.9% sterile saline. Animals were sacrificed at 2 h post injection of the radioactive probe, and major organs were collected, weighed, and counted in a Wizard^2^ automatic γ-counter (PerkinElmer, Boston, MA). The radiopharmaceutical uptake was expressed as a percentage of injected dose per gram (%ID/g) using the following formula: [(activity in the target organ/grams of tissue)/injected dose] × 100%.

### General PET/CT Imaging

All PET/CT images (*n* = 90) were acquired on an Inveon PET/CT (Siemens) and reconstructed scans were analyzed using the Inveon Research Workplace Software (Siemens). To acquire PET/CT images, animals were anesthetized with 2% isoflurane and positioned on the scanner bed. Animals were intravenously injected with 200–300 µCi of [^18^F]PARPi in 100–200 μL 10% EtOH in 0.9% sterile saline. Except for the determination of tracer kinetics, [^18^F]PARPi was allowed to clear for 2 h prior to PET/CT imaging. PET data were collected for 5–10 min, followed by CT.

### PET/CT imaging agent kinetics

JHU-LX48 SCLC PDX tumor bearing NSG mice (*n* = 3/group, 3 groups, 9 mice total) were administered 200–300 µCi of [^18^F]PARPi and imaged at 30, 60, or 120 min post injection. Different mice were used for each group to eliminate, as much as possible, the influence of anesthesia on tracer distribution. Activity concentration was quantified by averaging the mean values taken from VOIs drawn on the chosen organs as they appeared in the CT and reported as a mean %ID/g ± SD.

### PET/CT tumor size study

JHU-LX48 SCLC PDX cells where implanted into NSG mice (*n* = 6 mice) 7 days prior to the first imaging. [^18^F]PARPi PET/CT imaging was carried on the day of tumor implantation and once every 7 days thereafter. This was continued for 8 consecutive weeks until mice were sacrificed to avoid surpassing a tumor size limit of 1500 mm^3^. Activity concentration was quantified by averaging the mean or max values taken from VOIs drawn over the entire tumor as it appeared in the CT and portrayed as mean and max %ID/g ± SD.

### PET/CT dosing study

JHU-LX48 SCLC PDX tumor bearing mice (*n* = 3/group, 9 groups, 27 mice total) were administered varying doses of a therapeutic inhibitor via tail vein injection to serve as blocking agents prior to imaging. The doses administered for olaparib were 50 mg/kg, 15 mg/kg, 5 mg/kg and 1.5 mg/kg. The doses administered for talazoparib were 15 mg/kg, 5 mg/kg, 0.5 mg/kg and 0.3 mg/kg. The control group received no therapeutic inhibitor. [^18^F]PARPi was administered 30 min after the blocking doses and imaging was performed as described above. Activity concentration was quantified by averaging the mean values taken from VOIs drawn over the entire tumor as it appeared in the CT and portrayed as a mean %ID/g ± SD.

### In vivo kinetics

JHU-LX48 SCLC PDX tumor bearing mice (*n* = 3/group, 15 groups, 45 mice total) were administered therapeutic doses of olaparib (50 mg/kg) or talazoparib (0.3 mg/kg) via tail vein injection to serve as blocking agents prior to imaging. The amount of time in between administration of the blocking agent and tracer was 1, 2, 4, 8, 24, or 48 h. After allowing this clearance time for the blocking agent, the imaging was performed as described above. Activity concentration was quantified by averaging the mean values taken from VOIs drawn over the entire tumor as it appeared in the CT and portrayed as a mean %ID/g ± SD.

### Autoradiography

JHU-LX48 SCLC PDX tumor bearing mice (*n *= 3/group, 3 groups, 9 mice total) were injected either with 200–300 μCi of [^18^F]PARPi (i.v.) alone, or with a previous injection of talazoparib (0.3 mg/kg) or olaparib (50 mg/kg) 30 min prior to [^18^F]PARPi. 2 h post [^18^F]PARPi injection, mice were sacrificed and tumor and muscle were resected, immediately frozen in OCT compound and 10 µm cryosections were prepared using a cryotome. Sections from 3 different tumor areas from each tumor (*n* = 3/group) were exposed to an autoradiography plate overnight and read the next day. ImageJ was used to quantify the signal intensity of each tissue. Adjacent sections were used for PARP1 IHC and H&E staining to confirm tissue identity and PARP1 expression.

### Statistical analysis

Data are expressed as mean ± SD. Statistical analyses were performed with GraphPad Prism, Version 7.0a (La Jolla, CA). Non-parametric, two-tailed Student’s *t*-tests with assumption of unequal standard deviations were used to calculate statistics. *P-*values < 0.05 were considered significant.

### Data availability

All the data supporting the findings of this study are available within the article and its supplementary information files or from the corresponding author upon reasonable request.

## Electronic supplementary material

Supplementary Information
