# Supplementary material for: Target engagement imaging of PARP inhibitors in small-cell lung cancer
Source: Nat Commun. 2018 Jan 12;9:176. doi: 10.1038/s41467-017-02096-w (PMC5766608; doi:10.1038/s41467-017-02096-w)
Supplement: Supplementary file 1 — Supplementary Information [file 41467_2017_2096_MOESM1_ESM.pdf]

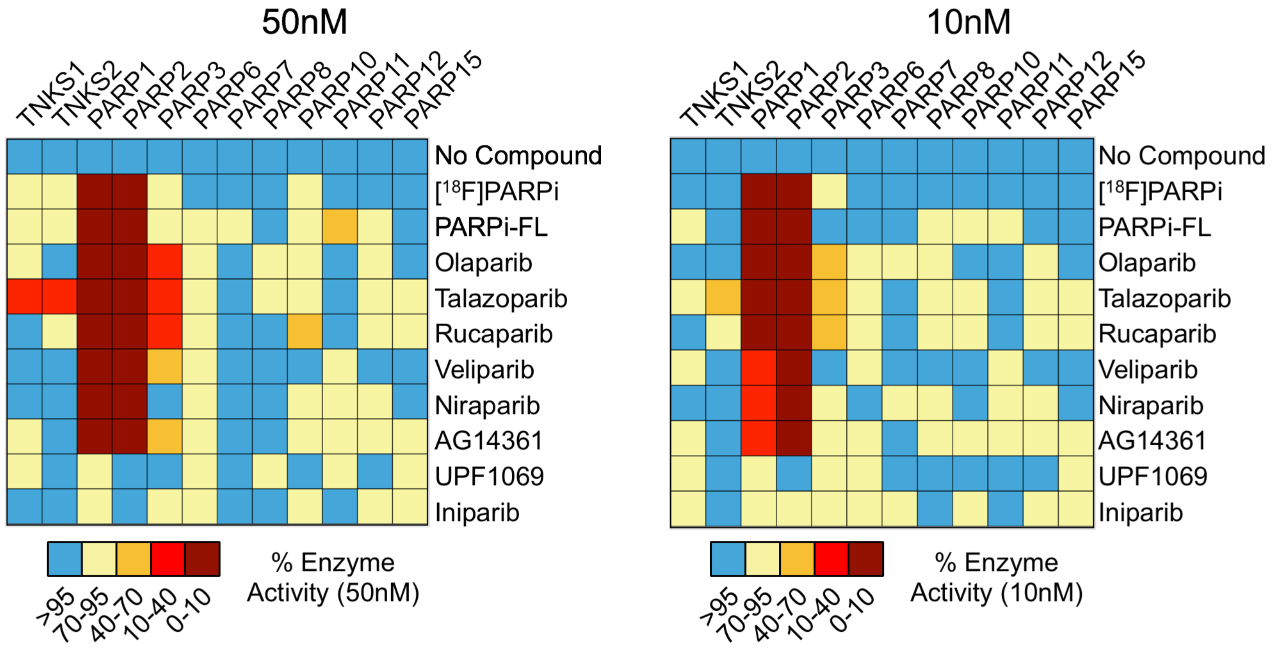

Supplementary Figure 1 | PARP enzyme family binding assays at additional concentrations. Data produced by BPS Bioscience, San Diego, CA, through a colorimetric Strep-HRP assay with a histone substrate and biotin-labeled NAD+ at 100 nM (Fig. 1b), 50 nM (left) and 10 nM (right) for each inhibitor and each enzyme.

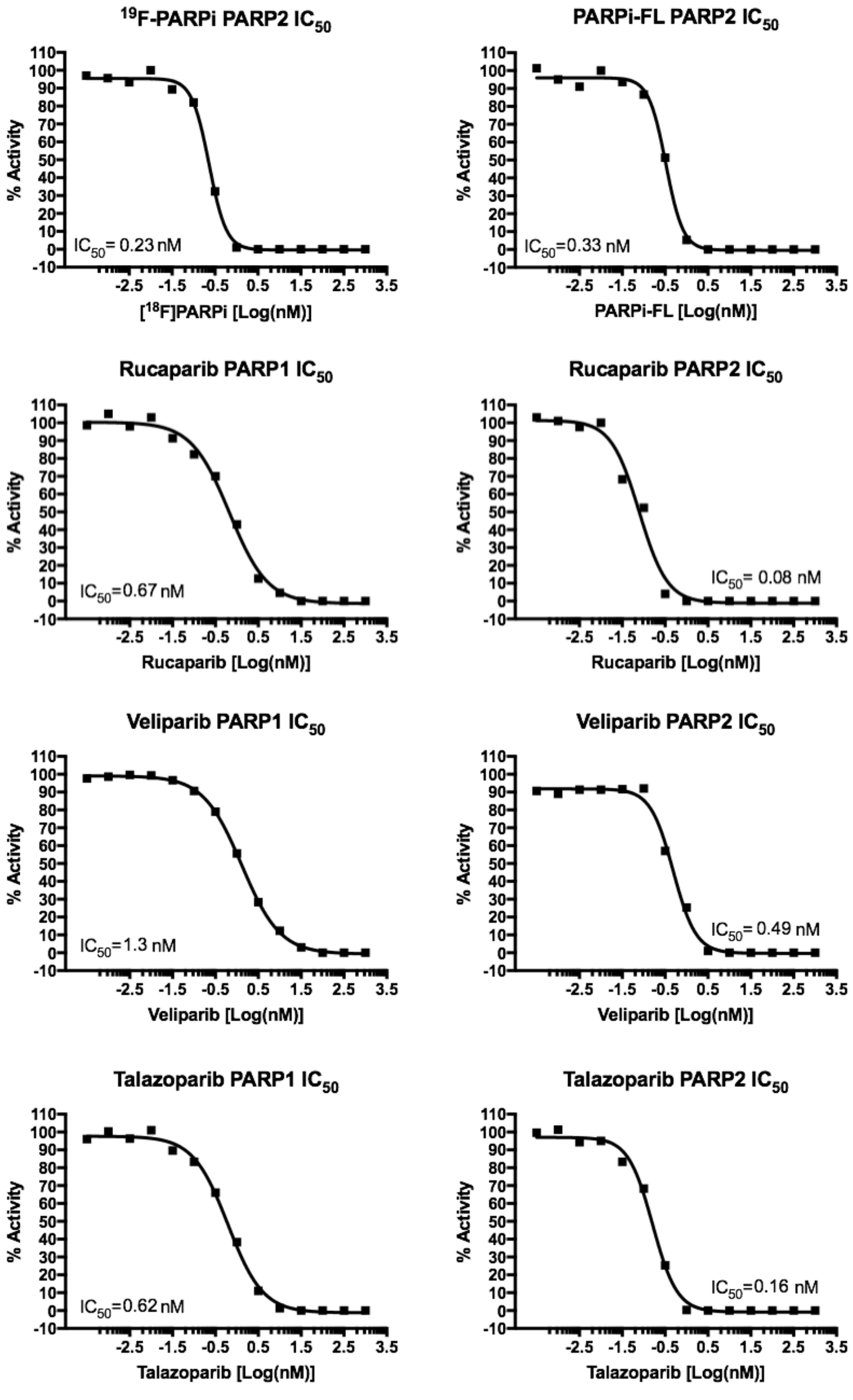

Supplementary Figure 2 | PARP1 and PARP2 IC_{50} values for selected inhibitors. Data produced by BPS Bioscience, San Diego, CA, through a colorimetric Strep-HRP assay with a histone substrate and biotin-labeled NAD+ at ten different concentrations ranging from 0.3 pM to 1 µM.

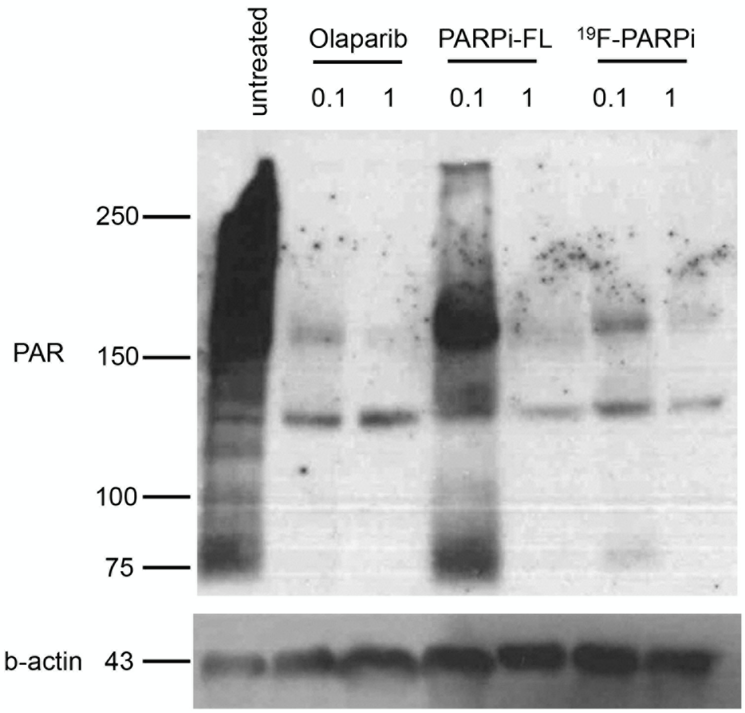

Supplementary Figure 3 | PARylation inhibition assay. To show that our PARP imaging agents PARPi-FL and [^{18}F]PARPi quantify PARP inhibition, we compared their ability to inhibit PARylation in comparison with olaparib. Both olaparib and ^{19}F-PARPi showed nearly complete inhibition at 0.1 μM after 30 min incubation time while PARPi-FL showed reduced PAR signal, but not complete inhibition. At 1 μM, the PAR signal for all three compounds is nearly completely eliminated.

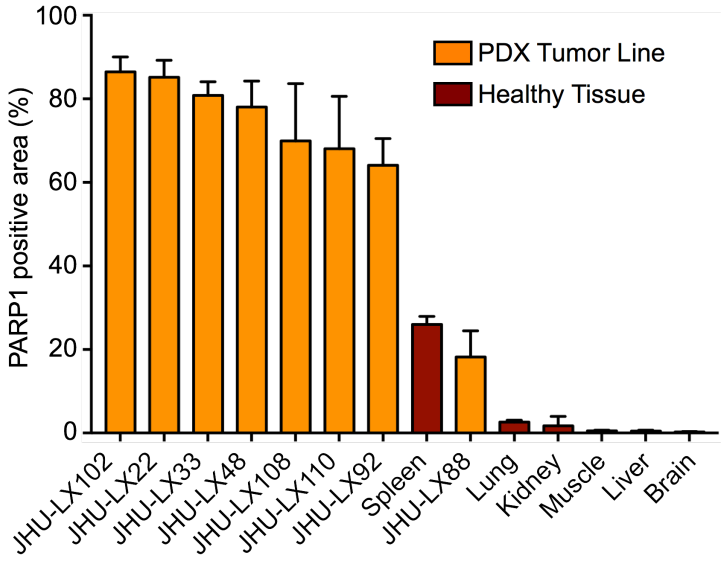

Supplementary Figure 4 | Quantification of PARP1 expression in PDX cell lines and other organs. PARP1 expression was calculated for 8 PDX cell lines including 7 SCLC lines (JHU-LX102, JHU-LX22, JHU-LX33, JHU-LX48, JHU-LX108, JHU-LX110, JHU-LX92) and 1 lung squamous cell carcinoma lung cancer line (JHU-LX88) via tissue microarrays (TMAs). Also included were samples from 6 healthy tissues. TMAs were stained for PARP1 and positive area was calculated for each tissue core using an automated color thresholding protocol on digitalized slides. The cores were evaluated for each PDX model and error bars represent the standard deviation (SD).

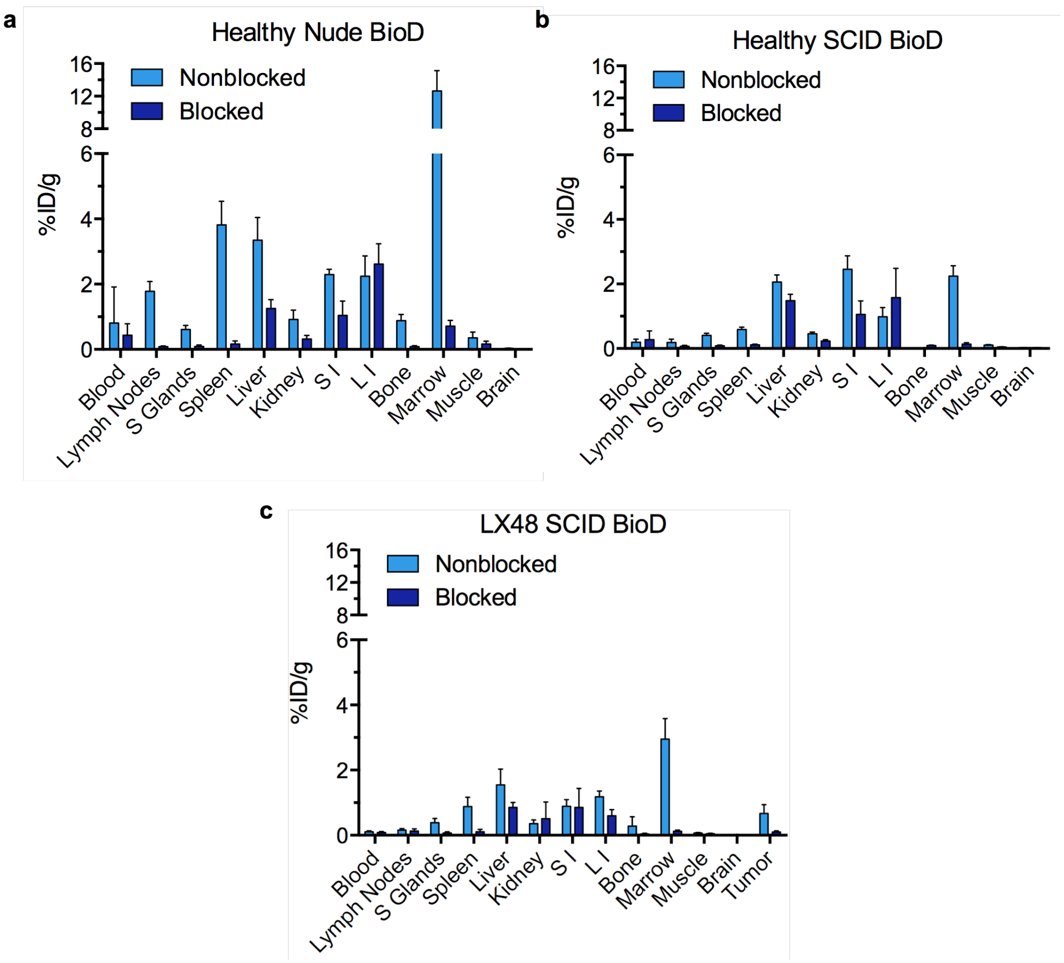

Supplementary Figure 5 | Biodistribution studies in nude mice and NSG mice. After injection of [^{18}F]PARPi into either (**a**) healthy nude mice, (**b**) healthy NSG mice, or (**c**) NSG mice bearing subcutaneous JHU-LX48 SCLC PDX tumors, mice (n=6/group) were euthanized at 2 h post-injection, organs were collected and activity counted. Radioactivity and organ weights were measured. Displayed are means and error bars represent the standard deviation (SD).

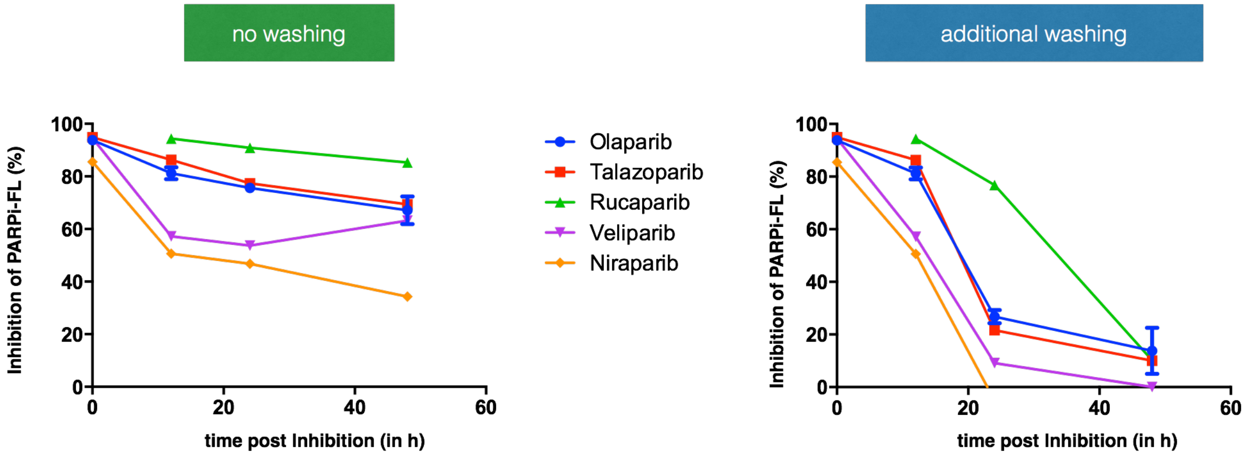

Supplementary Figure 6 | Binding kinetics of PARP1 inhibitors *in vitro*. The half-life of PARP1 inhibitors *in vitro* is dependent on washing regimes. After incubation, cells were either left in the same medium for the entire post-incubation time or additional media exchanges were performed after 6, 20 and 40 h. Subsequently, cells were incubated with PARPi-FL and its uptake was determined by flow cytometry. While overall half-lives are subject to the amount of media exchanges, trends within the panel of inhibitors persisted.

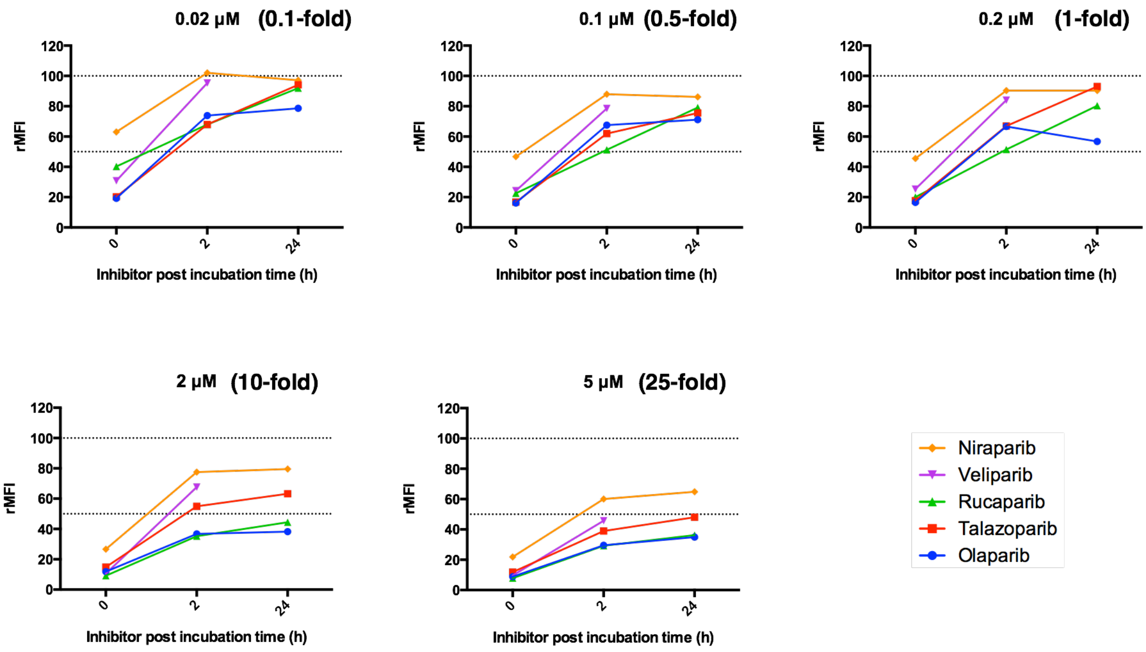

Supplementary Figure 7 | Effect of varying PARP inhibitor concentrations on PARPi-FL binding recovery. The recovery of PARPi-FL binding changed depending on the PARP inhibitor concentration. Cells were incubated first with 0.02, 0,1, 0,2, 2, 5 or 12.5 µM PARP inhibitor for 25 min at 37 °C, then washed and supplied with fresh media for the post incubation time of 2 or 24 h. Then, cells were incubated with 0.2 µM PARPi-FL for 15 min at 37 °C, followed by a 10 min wash with medium. PARPi-FL fluorescence was analyzed using flow cytometry. Recovery of the PARPi-FL signal after 24 h decreases with increasing PARP inhibitor concentrations, especially at 2 and 5 µM, while initial blocking of PARPi-FL at 0 h is decreased at the lowest concentrations.

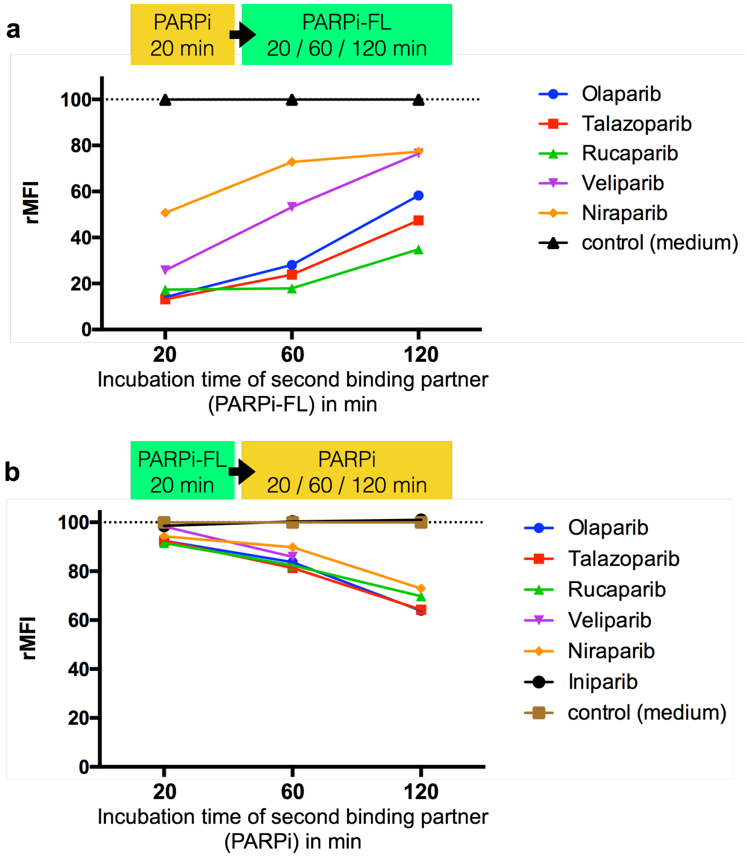

Supplementary Figure 8 | Target engagement versus differences in affinity. To confirm that the measured in vitro kinetics depend on target engagement and not merely reflect differences in affinity of the different PARP inhibitors, it was determined whether there was equilibrium binding between a PARP inhibitor and PARPi-FL. Therefore, cells were (**a**) either first incubated with PARPi-FL for 20min, followed by a PARP inhibitor for 20, 60 or 120 min or (**b**) first with one of the PARP inhibitors for 20 min, followed by PARPi-FL for 20, 60 or 120 min. Fluorescence uptake per cell was analyzed using flow cytometry. At the shortest incubation time (20 min), the second binding partner cannot bind to its target because it is already occupied by the first binding partner, independent of the differences in affinity reported in Fig. 1. A change toward equilibrium binding is only observed if the second binding partner was incubated for 2 hours, indicating a slow k_{off} rate of the PARP1 inhibitors. This corroborates that when 20-30 min binding protocols are used, target engagement can be quantified because at these short incubation times the second binding partner cannot compete the first binding partner off.

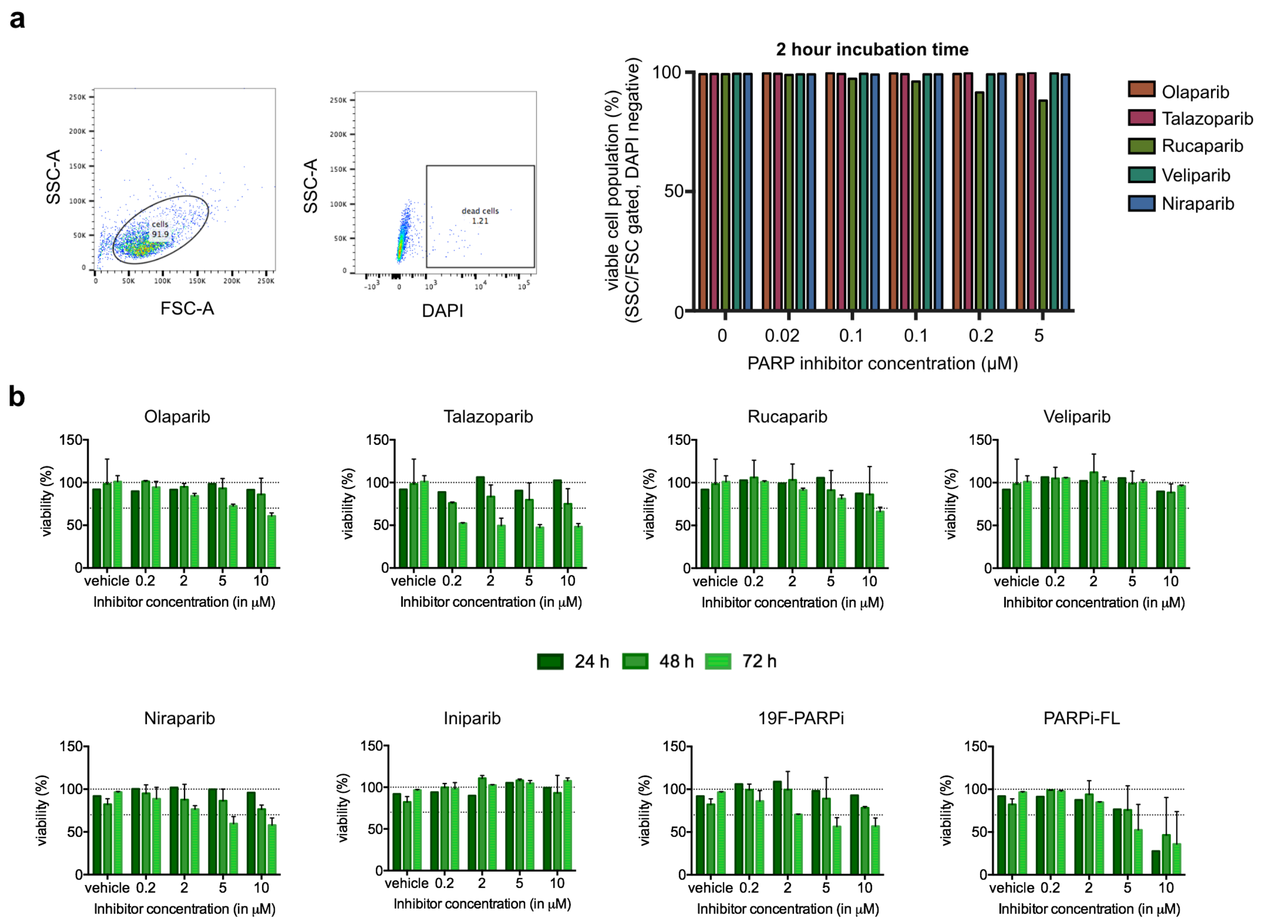

c

Inhibitor	MW in g/mol	IC50 (96 h)
Olaparib O	435	8.2 µM
Talazoparib T	380.12	0.16 µM
Rucaparib R	421.36	9.9 µM
Veliparib V	244.29	88.8 µM
NIraparib N	320.39	4.6 µM
Iniparib	292.04	> 100 µM
18F/19F-PARPi	488.5	3.7 µM
PARPi-FL	650	4.8 µM

Supplementary Figure 9 | *In vitro* toxicity of the tested PARP inhibitors and imaging tracers on JHU-LX22 cells. (a) Assessment of viable cell population during flow cytometry after a 2 hour incubation time of several PARP inhibitors at concentrations between 0 and 5 µM. (b) Cell viability determination using Alamar Blue assays. Cells were incubated with 0, 0.2, 2, 5 or 10 µM of each compound for 24, 48, 72 or 96 h and cell viability was assessed using the Alamar Blue assay. (c) IC_{50} values derived from Alamar Blue assays at 72 h and 96 h. PARPi-FL and ^{19}F-PARPi showed very similar effects on JHU-LX22 cell viability as other inhibitors. Viability data represent means from three independent experiments, each with four parallels of each sample. Error bars represent the standard error (SEM).

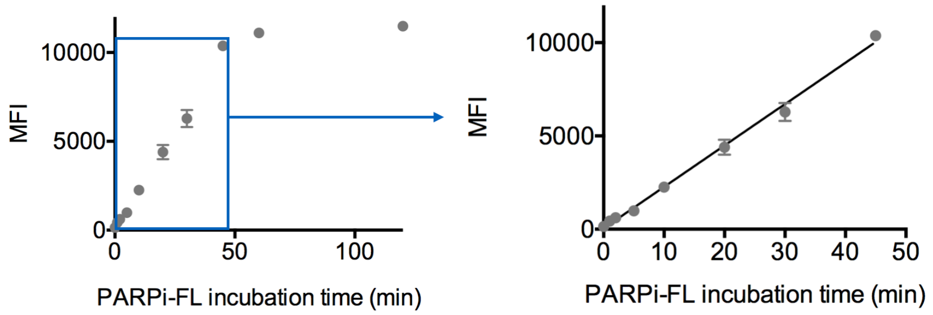

Supplementary Figure 10 | PARPi-FL uptake at different incubation times. PARPi-FL uptake over time was measured between 1 min and 120 min.
